# Supplementary material for: Targeting lysyl oxidase (LOX) overcomes chemotherapy resistance in triple negative breast cancer
Source: Nat Commun. 2020 May 15;11:2416. doi: 10.1038/s41467-020-16199-4 (PMC7229173; doi:10.1038/s41467-020-16199-4)
Supplement: Supplementary file 1 — Supplementary Information [file 41467_2020_16199_MOESM1_ESM.pdf]

SUPPLEMENTARY INFORMATION

Targeting lysyl oxidase (LOX) overcomes chemotherapy resistance in triple negative breast cancer

Saatci et al.

Supplementary Figures

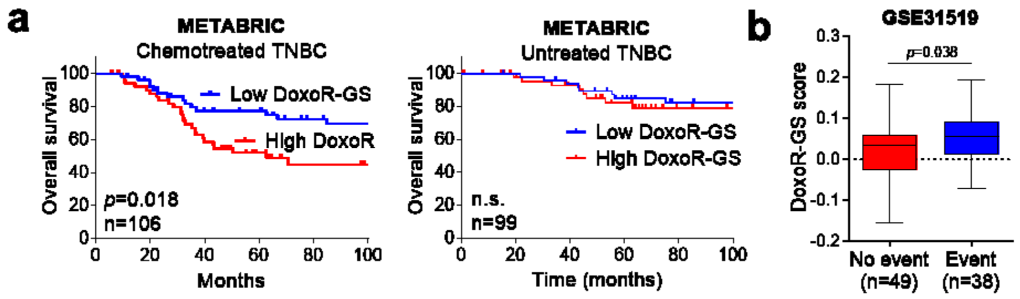

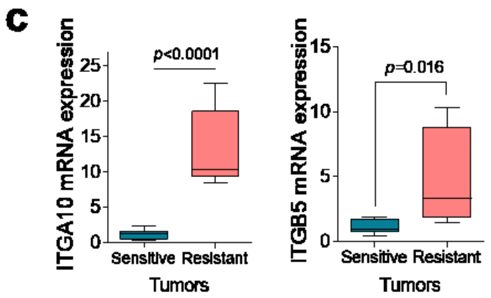

d

KM-Plotter - Breast cancer relapse free survival analysis for ITGA10

	Chemotherapy					
	-			+		
Subtype	n	p-value	HR	n	p-value	HR
Basal	184	0.48	0.84	132	0.53	0.82
Luminal A	1098	0.03	0.77	83	0.77	1.15
Luminal B	548	0.14	0.81	145	0.007	0.45
HER2+	43	0.92	1.05	61	0.60	0.81

e

KM-Plotter - Breast cancer relapse free survival analysis for ITGB5

	Chemotherapy					
	-			+		
Subtype	n	p-value	HR	n	p-value	HR
Basal	184	0.92	0.98	132	0.13	1.63
Luminal A	1098	0.14	0.84	83	0.94	1.04
Luminal B	548	0.09	0.79	145	0.87	1.05
HER2+	43	0.18	2.09	61	0.02	2.63

Supplementary Figure 1. Association of DoxoR-GS and integrins with chemoresistance in TNBCs. a. Kaplan-Meier survival curves representing percentage overall survival (OS) in chemotherapy-treated (n=106) and untreated (n=99) TNBC patients from METABRIC based on low vs. high (median) DoxoR-GS score. **(b)** Expression levels of DoxoR-GS score in patients from GSE31519 that has no event (n=49) vs. event (n=38), described as either relapse- or distant metastasis. **c.** qRT-PCR analysis showing the expression of *ITGA10* and *ITGB5* in doxorubicin sensitive (n=8) vs. resistant (n=6) tumors. **d, e.** Tables summarizing the results of Kaplan-Meier relapse-free survival (RFS) analysis based on ITGA10 (d) and ITGB5 (e) expression (median) in patients representing different breast cancer subtypes that received or did not receive chemotherapy. Data represents mean ± SD. In Box plots, the box depicts median, 25th to 75th percentiles, and the whisker depicts min to max. Two-sided Student's t-test was used to calculate statistical difference between two groups. Significance for survival analyses was calculated by log-rank (Mantel-Cox) test. n.s., not significant, HR: hazard ratio. Source data are provided as a Source Data file.

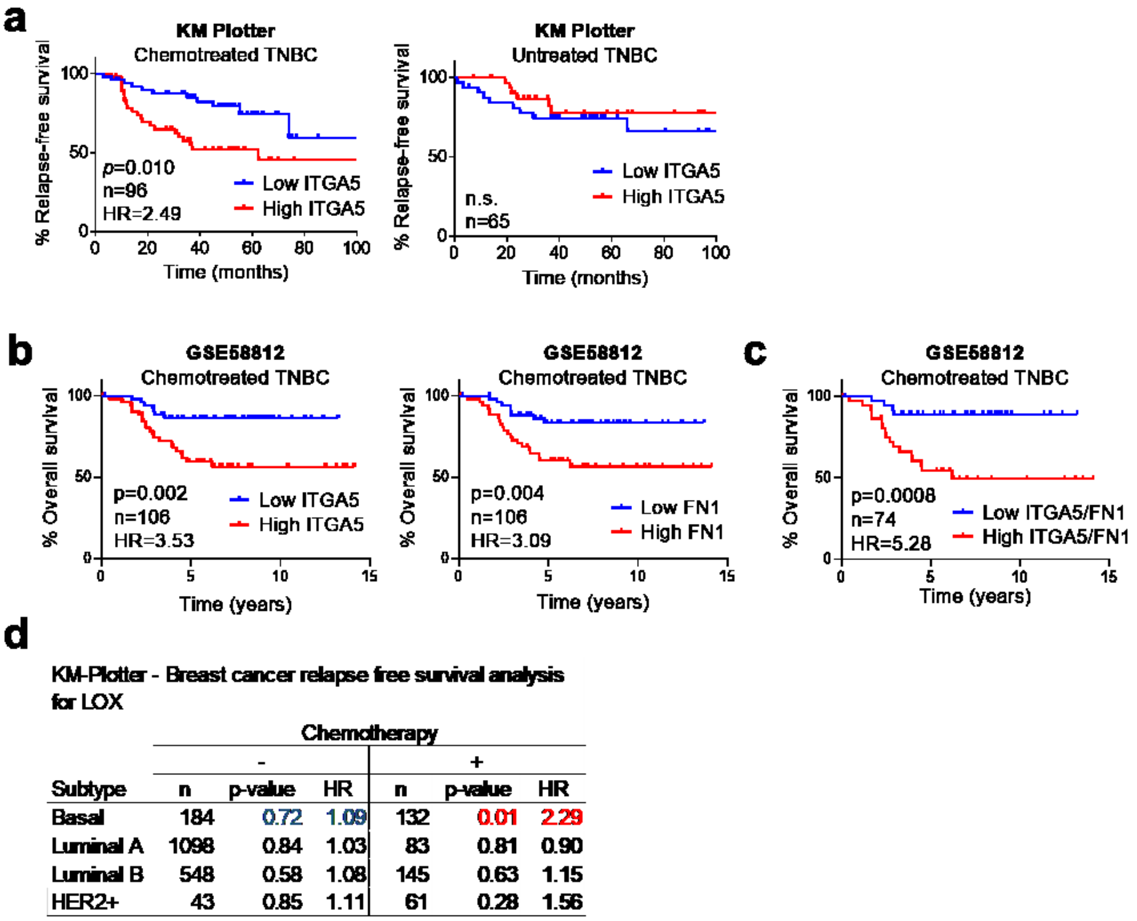

d

KM-Plotter - Breast cancer relapse free survival analysis for LOX

	Chemotherapy					
	-			+		
Subtype	n	p-value	HR	n	p-value	HR
Basal	184	0.72	1.09	132	0.01	2.29
Luminal A	1098	0.84	1.03	83	0.81	0.90
Luminal B	548	0.58	1.08	145	0.63	1.15
HER2+	43	0.85	1.11	61	0.28	1.56

Supplementary Figure 2. Association of ITGA5/FN1/LOX with prognosis in breast cancer. a. Kaplan-Meier survival curves representing the percentage RFS in chemotherapy-treated (n=96) and untreated TNBC (n=65) patients based on low vs. high (median) ITGA5 expression. **b.** Kaplan-Meier survival curves representing the percentage OS in chemotherapy-treated TNBC patients from GSE58812 that were separated based on ITGA5 and FN1 levels from median (n=106). **c.** Kaplan-Meier survival curves representing the percentage OS in chemotherapy-treated TNBC patients from GSE58812 that were separated based on combined ITGA5 and FN1 levels from median (n=74). **d.** Table summarizing LOX expression-based Kaplan-Meier RFS analysis of patients representing different breast cancer subtypes that received or did not receive chemotherapy. Significance for survival analyses was calculated by log-rank (Mantel-Cox) test. n.s., not significant, HR: hazard ratio. Source data are provided as a Source Data file.

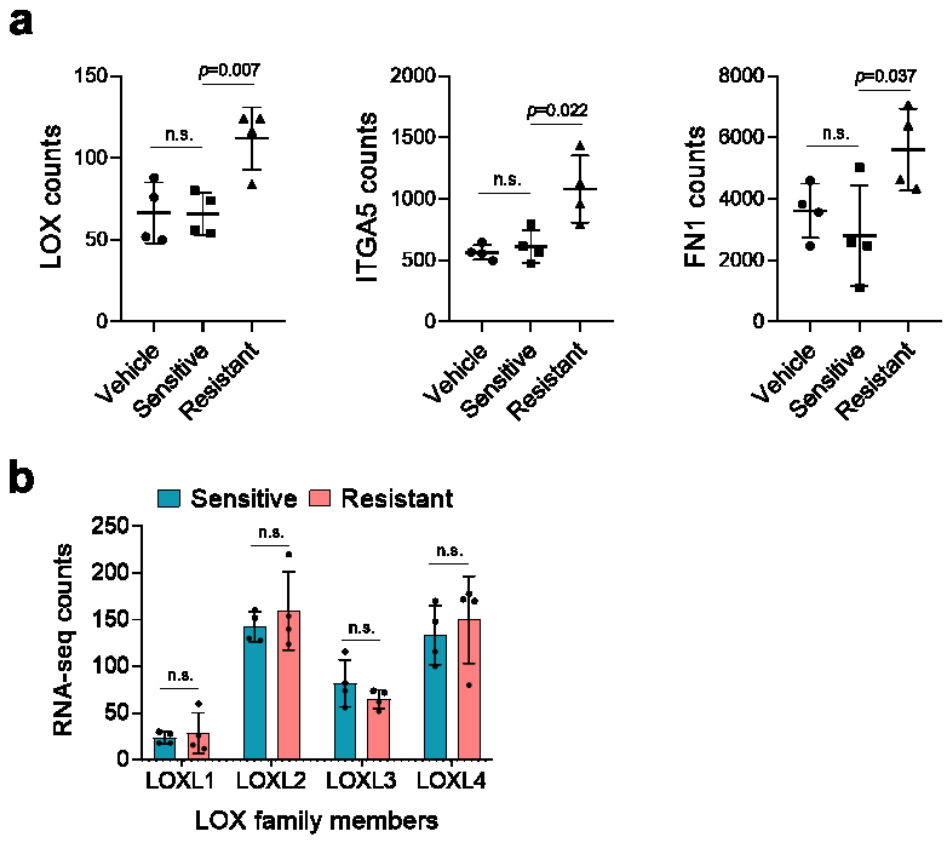

Supplementary Figure 3. Association of LOX family/ITGA5/FN1 expression with chemoresistance. a. RNA-Seq read counts of LOX, ITGA5 and FN1 in vehicle-treated, doxorubicin-sensitive and -resistant MDA-MB-231 tumors (n=4). **b.** Bar-graphs showing the expression of LOX family members from the RNA-Seq data of doxorubicin-sensitive (n=4) vs. doxorubicin-resistant (n=4) tumor xenografts. Data represents mean ± SD. Two-sided Student's t-test was used to calculate statistical difference between two groups. n.s., not significant. Source data are provided as a Source Data file.

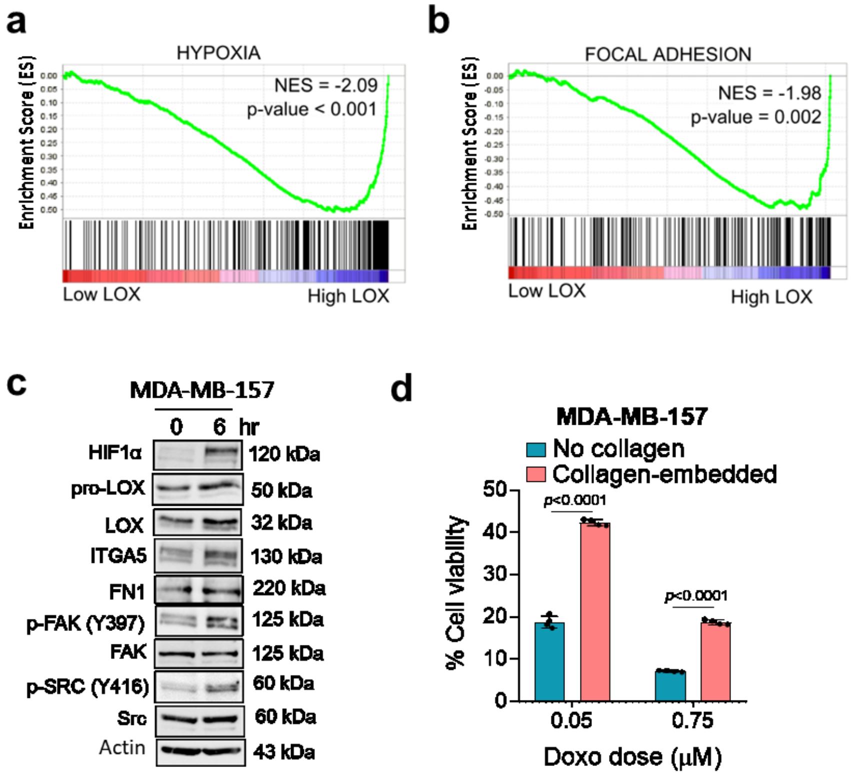

Supplementary Figure 4. Association of LOX with hypoxia and FA and hypoxic regulation of LOX/ITGA5/FN1 in MDA-MB-157. a, b. Enrichment plots for genes involved in hypoxia (a) and focal adhesion (b) in chemotherapy-treated TNBC patients from GSE58812 (n=106) with low vs. high (median) LOX expression. **c.** Western blot analysis of LOX, ITGA5, and FN1 and the downstream markers in MDA-MB-157 cells cultured in hypoxic conditions. **d.** Doxorubicin response of MDA-MB-157 cells cultured with or without type I collagen for 72 hours (n=4). Data represents mean ± SD. Two-sided Student's t-test was used to calculate statistical difference between two groups. NES: normalized enrichment score. Source data are provided as a Source Data file.

a

GSE31519

	event	no event
High LOX	25	19
Low LOX	13	30

Chi-square testing p=0.012

b

GSE25066

	RD	pCR
High LOX	63	20
Low LOX	50	37

Chi-square testing p=0.011

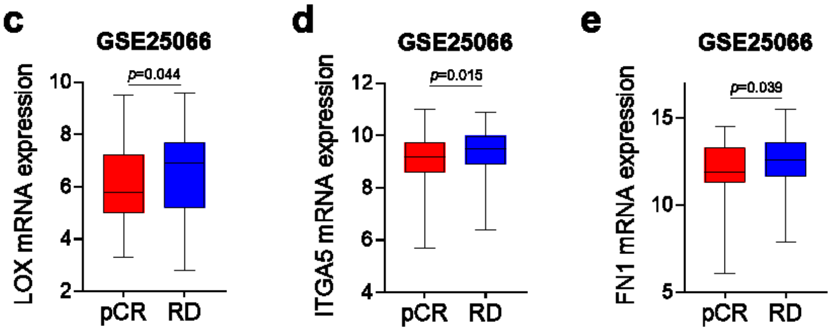

Supplementary Figure 5. Association of LOX with disease progression in chemotreated-TNBC patients. a, b. The association of LOX expression with disease progression and therapy response using GEO databases, GSE31519 (a) and GSE25066 (b). Number of patients in each group is provided, and the statistical testing was performed by chi-square testing. **c-e.** Levels of LOX, ITGA5 and FN1 in patients with pathologic complete response (pCR, n=57) vs. residual disease (RD, n=113) from GSE25066. Data represents mean ± SD. In Box plots, the box depicts median, 25th to 75th percentiles, and the whisker depicts min to max. Two-sided Student's t-test was used to calculate statistical difference between two groups. Source data are provided as a Source Data file.

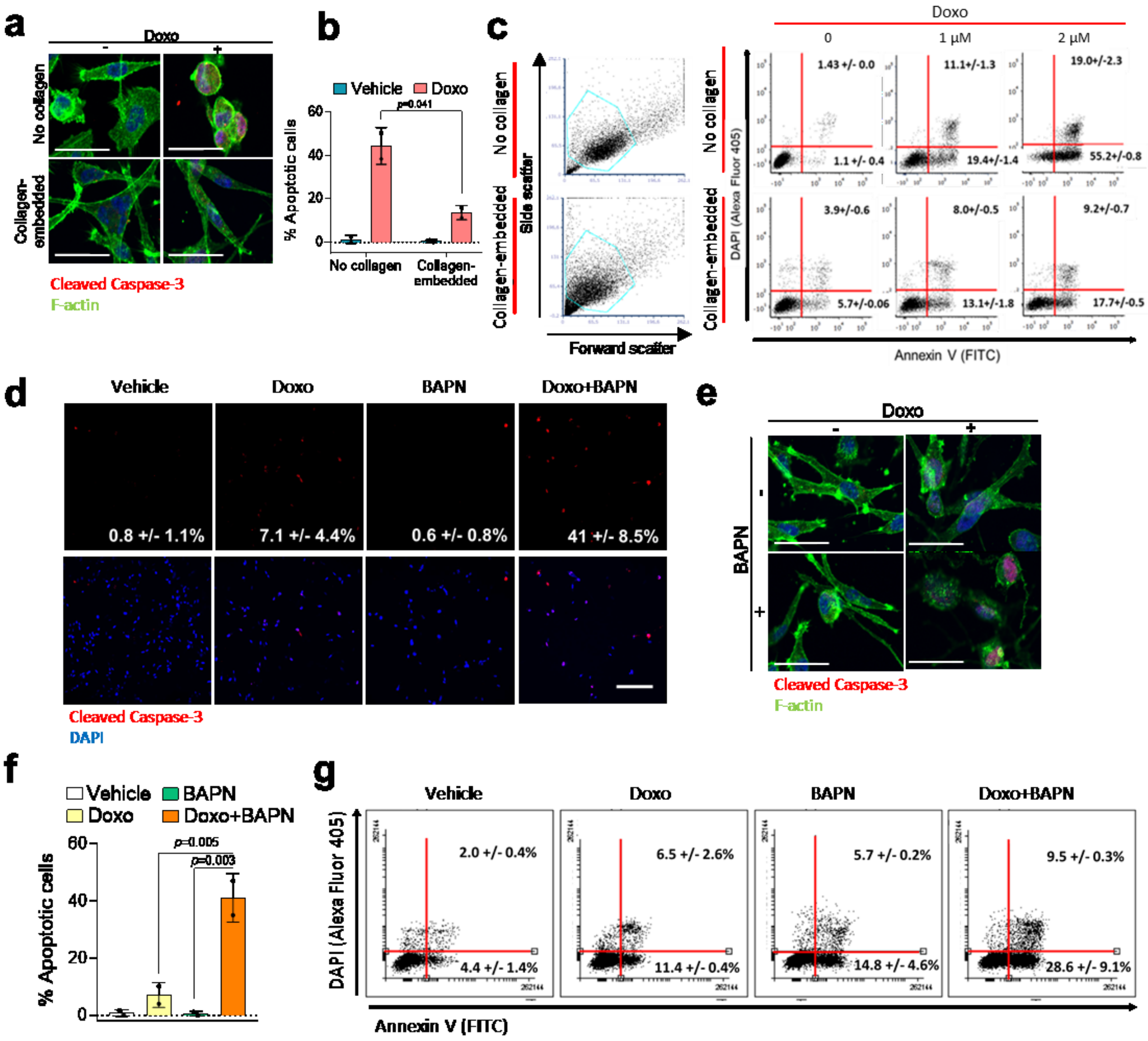

Supplementary Figure 6. Apoptosis assessment upon collagen-embedding. a, b. Cleaved Caspase-3 (red) and phalloidin (green) staining (a) and the percentage of Cleaved Caspase-3 positive cells (b) under doxorubicin treatment in the absence or presence of collagen type I (n=2). **c.** Dot plots showing the percentage of single or double positive cells for Annexin V and DAPI (right panel) (n=2). Gating strategy was provided on the left panel. **d.** Cleaved Caspase-3 (red) and DAPI (blue) staining of single agent- vs. combination-treated cells. Numbers on the right corner show the % apoptotic cells, n=2. **e, f.** Cleaved Caspase-3 (red) and phalloidin (green) staining (e) and the percentage of Cleaved Caspase-3 positive cells (f) under combination treatment in the presence of type I collagen (n=2). **g.** Dot plots showing the percentage of single or double positive cells for Annexin V and DAPI under doxorubicin and BAPN treatment, alone or in combination (n=2). Data represents mean ± SD. Two-sided Student's t-test was used

to calculate statistical difference between two groups. One-way ANOVA with Dunnett's test was performed to compare mean of combination-treated group with single agent treatments in **f**. Scale bar=50 µm for **a**, **d** and **e**. Source data are provided as a Source Data file.

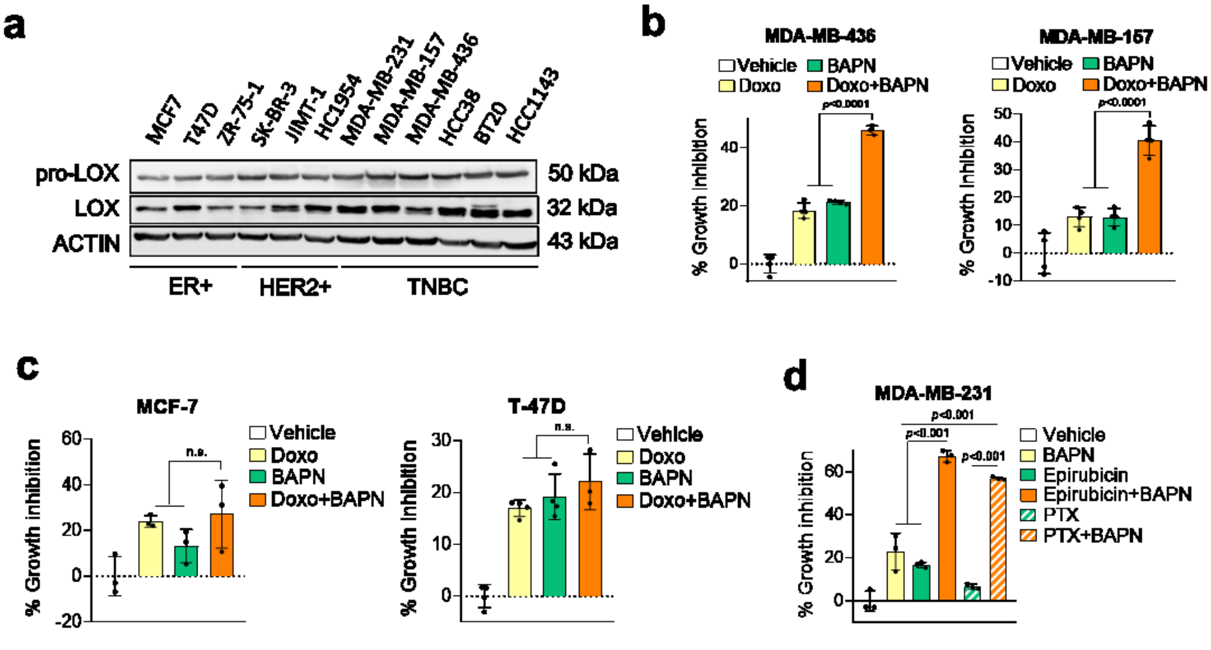

Supplementary Figure 7. Association of LOX with chemoresponse in breast cancer subtypes. a. Western blot analysis of LOX and pro-LOX in a panel of breast cancer cell lines. **b.** % growth inhibition upon Doxo+BAPN treatment of collagen I-embedded MDA-MB-436 (left panel) and MDA-MB-157 cells (right panel) (n=3-4). **c.** % growth inhibition upon Doxo+BAPN treatment of type I collagen-embedded ER+ models (n=3-4). **d.** % growth inhibition upon paclitaxel (PTX) or epirubicin treatment in combination with BAPN in collagen I-embedded MDA-MB-231 cells (n=3). Data represents mean ± SD. One-way ANOVA with Dunnett's test was performed to compare mean of combination-treated group with single agent treatments in **b-d**. Source data are provided as a Source Data file.

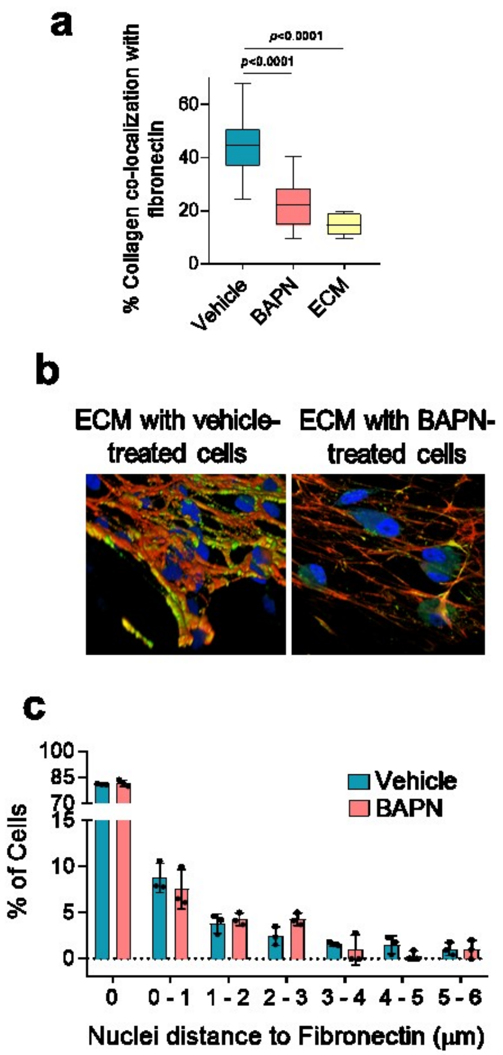

Supplementary Figure 8. Collagen/fibronectin co-localization and cell contact upon LOX inhibition. **a.** Co-localization of collagen fibers with fibronectin in HFF-derived ECM from vehicle (n=30) vs. BAPN-treated (n=25) MDA-MB-231 cells. Only ECM (n=9) group represents the staining of type I collagen and fibronectin in the absence of MDA-MB-231 cancer cells. **b.** 3D images of HFF-derived ECM showing the contact of cancer cells with the surrounding ECM. **c.** The percentage of cells having a range of predefined distances of cell nuclei to the surrounding ECM fibers. A distance of '0' indicates direct contact (n=3). Data represents mean ± SD. In Box plots, the box depicts median, 25th to 75th percentiles, and the whisker depicts min to max. Two-sided Student's t-test was used to calculate statistical difference between two groups. Source data are provided as a Source Data file.

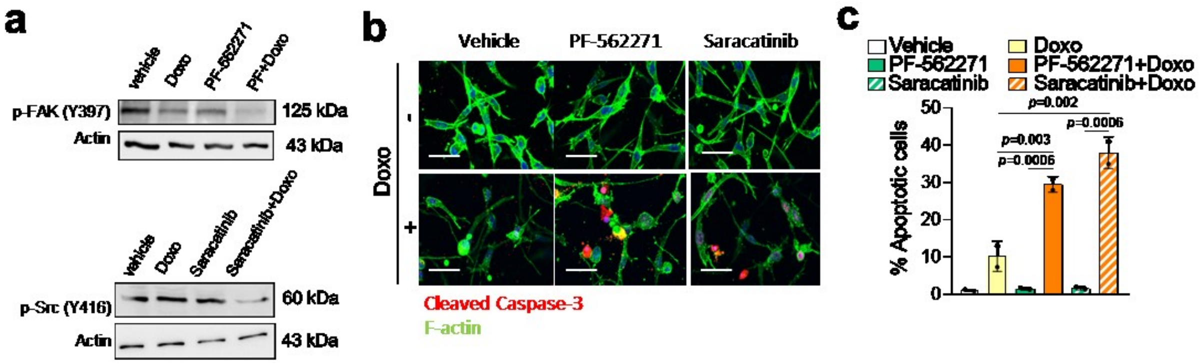

Supplementary Figure 9. Change in FAK/Src signaling and apoptosis upon Doxo+FAK/Src inhibition. a. Western blot analysis of p-FAK and p-Src levels upon treatment of collagen I-embedded MDA-MB-231 cells with doxorubicin in combination with PF-562271 or Saracatinib. **b, c**. Cleaved Caspase-3 (red) and F-actin (green) staining (b) and the quantification of apoptotic fraction (c) in collagen I-embedded MDA-MB-231 cells treated with PF-532271 or Saracatinib in combination with doxorubicin for 48 hours (n=2). Data represents mean ± SD. One-way ANOVA with Dunnett's test was performed to compare mean of combination-treated group with single agent treatments in **c**. Scale bar= 50 µm. Source data are provided as a Source Data file.

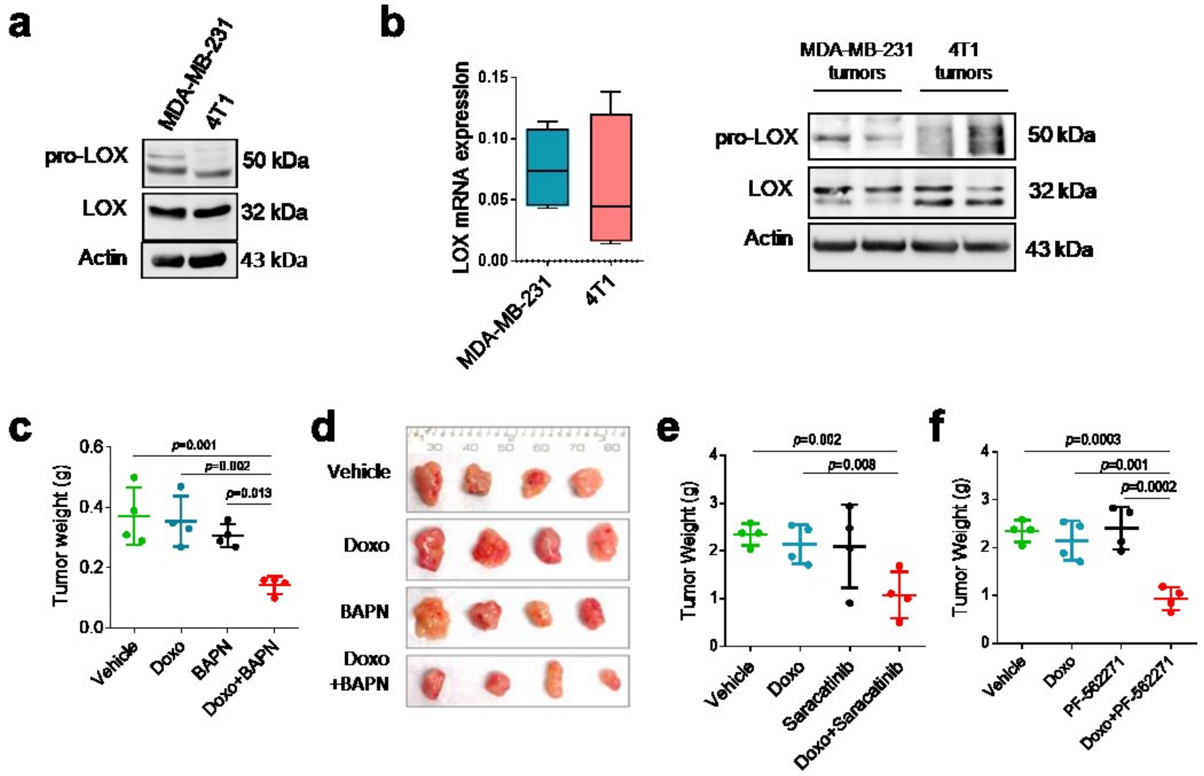

Supplementary Figure 10. LOX expression in 4T1 model and tumor weights upon doxorubicin sensitization. a. Western blot analysis of LOX in MDA-MB-231 and 4T1 cells. **b.** qRT-PCR (n=9) and western blot analysis of LOX in MDA-MB-231 and 4T1 tumors. **c.** Change in tumor weight in 4T1 tumors in Balb/c mice upon treatment with doxorubicin and BAPN (n=4). **d.** Images of tumors isolated from the mice in c. **e, f.** Change in tumor weight in 4T1 tumors in Balb/c mice upon treatment with doxorubicin and Saracatinib (e) or PF562271(f) (n=4). Data represents mean ± SD. In the Box plot, the box depicts median, 25th to 75th percentiles, and the whisker depicts min to max. One-way ANOVA with Dunnett's test was performed to compare mean of combination-treated group with single agent treatments in **c**, **e** and **f**. Source data are provided as a Source Data file.

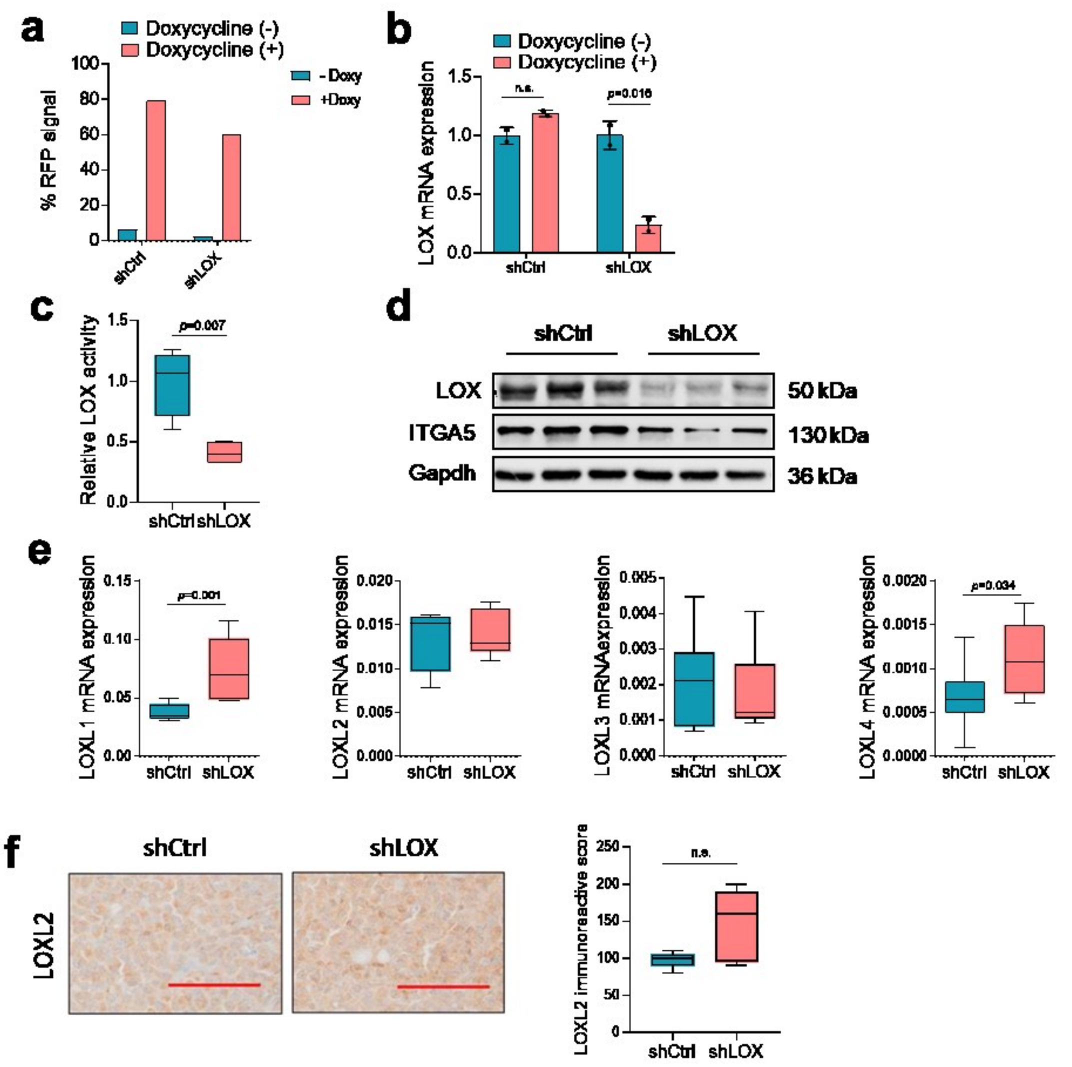

Supplementary Figure 11. Expression of LOX family members upon LOX knockdown *in vivo*. a. Validation of the induction of the red fluorescent protein (RFP)-labelled shRNA expression by measuring RFP signal upon doxycycline treatment. **b.** qRT-PCR analysis of LOX in MDA-MB-231 cells after induction of shRNA upon doxycycline treatment (n=2). **c**. Relative LOX activity in tumors that were collected at the end of the experiment (n=4). **d.** Western blot analysis of LOX and ITGA5 in shCtrl vs. shLOX tumors. **e.** Expression of other LOX family members in shCtrl vs. shLOX tumors (n=9). **f.** IHC staining and quantification of LOXL2 in shCtrl vs. shLOX tumors (n=5). Data represents mean ± SD. In Box plots, the box depicts median, 25th to 75th percentiles, and the whisker depicts min to max. Two-sided Student's t-test was used to calculate statistical difference between two groups. Scale bar=100 µm. Source data are provided as a Source Data file.

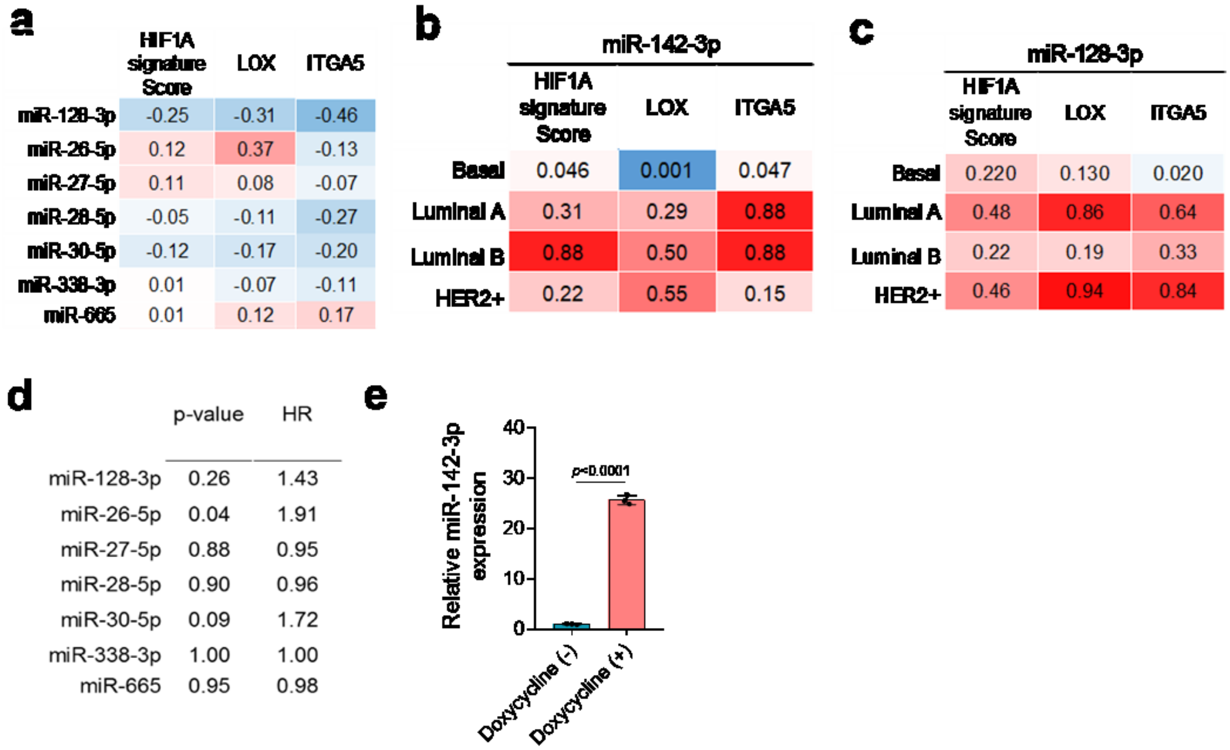

a

	HIF1A signature Score	LOX	ITGA5
miR-128-3p	-0.25	-0.31	-0.46
miR-26-5p	0.12	0.37	-0.13
miR-27-5p	0.11	0.08	-0.07
miR-28-5p	-0.05	-0.11	-0.27
miR-30-5p	-0.12	-0.17	-0.20
miR-338-3p	0.01	-0.07	-0.11
miR-665	0.01	0.12	0.17

b

	miR-142-3p		
	HIF1A signature Score	LOX	ITGA5
Basal	0.046	0.001	0.047
Luminal A	0.31	0.29	0.88
Luminal B	0.88	0.50	0.88
HER2+	0.22	0.55	0.15

c

	miR-128-3p		
	HIF1A signature Score	LOX	ITGA5
Basal	0.220	0.130	0.020
Luminal A	0.48	0.86	0.64
Luminal B	0.22	0.19	0.33
HER2+	0.46	0.94	0.84

d

	p-value	HR
miR-128-3p	0.26	1.43
miR-26-5p	0.04	1.91
miR-27-5p	0.88	0.95
miR-28-5p	0.90	0.96
miR-30-5p	0.09	1.72
miR-338-3p	1.00	1.00
miR-665	0.95	0.98

Supplementary Figure 12. Patient data analyses of miRNAs predicted to target HIF1A/LOX/ITGA5. **a**. Summary table of the Pearson correlation coefficients between miRNAs and HIF1A gene signature, LOX and ITGA5 expression in patients from GSE19783. An intense blue color shows a stronger negative correlation. **b, c.** Summary of the p-values for the Pearson correlation analysis between miR-142-3p (b) and miR-128-3p (c) expression and HIF1A gene signature score, LOX and ITGA5 expressions. An intense blue color shows a stronger negative correlation. **d.** Summary table of the p-values and hazard ratios (HRs) for Kaplan-Meier overall survival analysis based on the expression of miRNAs in chemotherapy-treated TNBC patients (n=106) separated from median. Data were retrieved from METABRIC. **e.** qRT-PCR of miR-142-3p upon doxycycline induction in MDA-MB-231 xenografts inducibly expressing miR-142-3p (n=3). Data represents mean ± SD. Two-sided Student's t-test was used to calculate statistical difference between two groups. Significance for survival analyses was calculated by log-rank (Mantel-Cox) test. Source data are provided as a Source Data file.

Supplementary Tables

Supplementary Table 1. Gene Set Enrichment Analysis (GSEA) of RNA-Seq data of TNBC xenografts in patient datasets for hypoxia

signaling.

	Sensitive vs. Vehicle		Sensitive vs. Resistant	
**Name of the gene set**	**NES**	**p-value**	**NES**	**p-value**
HALLMARK_HYPOXIA	0.63	0.99	-2.25	< 0.001
HARRIS_HYPOXIA	0.89	0.70	-2.25	< 0.001
MANALO_HYPOXIA_UP	0.86	0.85	-1.85	< 0.001
PID_HIF1_TFPATHWAY	1.1	0.31	-1.9	< 0.001
WINTER_HYPOXIA_METAGENE	0.83	0.91	-1.9	< 0.001

Supplementary Table 2. IPA-based Upstream Regulator Analysis using RNA-seq data of chemoresistant TNBC xenografts.

Gene ID	Log2 FC	Prediction (based on expression direction)	Literature findings
ADM	1.68	Activated	HIF1A upregulates
ANGPTL4	1.22	Activated	HIF1A upregulates
BHLHE40	0.74	Activated	HIF1A upregulates
BNIP3	0.73	Activated	HIF1A upregulates
BNIP3L	1.12	Activated	HIF1A upregulates
C9orf9	1.29	Activated	HIF1A upregulates
CXorf40B	1.02	Activated	HIF1A upregulates
EGLN3	1.62	Activated	HIF1A upregulates
EREG	1.05	Activated	HIF1A upregulates
ERO1L	0.63	Activated	HIF1A upregulates
FN1	1.09	Activated	HIF1A upregulates
HILPDA	0.97	Activated	HIF1A upregulates
HK2	0.86	Activated	HIF1A upregulates
ITGA5	0.84	Activated	HIF1A upregulates
L1CAM	0.70	Activated	HIF1A upregulates
LOX	0.80	Activated	HIF1A upregulates
MMP1	0.88	Activated	HIF1A upregulates
NDRG1	1.23	Activated	HIF1A upregulates
NEK8	0.71	Activated	HIF1A upregulates
PFKFB3	1.26	Activated	HIF1A upregulates
PFKFB4	0.80	Activated	HIF1A upregulates
PGF	0.79	Activated	HIF1A upregulates
PGK1	0.84	Activated	HIF1A upregulates
PLAC8	0.69	Activated	HIF1A upregulates
PPFIA4	0.95	Activated	HIF1A upregulates
PTGS2	1.57	Activated	HIF1A upregulates
SLC2A1	0.91	Activated	HIF1A upregulates
SLC2A3	1.26	Activated	HIF1A upregulates
AKAP12	1.15	Affected	HIF1A regulates
ANKRD37	0.74	Affected	HIF1A regulates
ANKZF1	0.63	Affected	HIF1A regulates
BRCA1	-1.43	Affected	HIF1A regulates
CITED2	0.86	Affected	HIF1A regulates
DNASE1	-1.03	Affected	HIF1A regulates
FAM13A	0.78	Affected	HIF1A regulates
IL17RA	0.65	Affected	HIF1A regulates
MMP9	-1.73	Affected	HIF1A regulates
TAF9B	0.62	Affected	HIF1A regulates
SSBP1	0.66	Inhibited	HIF1A downregulates

Supplementary Table 3. Clinical features of 77 TNBC cases treated with chemotherapy.

ID	Age	ER	PR	HER2	Grade	Morphology	ID	Age	ER	PR	HER2	Grade	Morphology
1	35	0	0	0	3	invasive ductal carcinoma	39	48	0	0	0	3	invasive ductal carcinomaa
2	51	0	0	0	3	NA	40	44	0	0	0	2	invasive ductal carcinoma
3	52	0	0	0	3	invasive ductal carcinoma	41	64	0	0	0	3	metaplastic carcinoma
4	58	0	0	0	NA	invasive ductal carcinomaa	42	59	0	0	0	3	invasive pleomorphic lobular carcinoma
5	77	0	0	0	3	invasive ductal carcinoma	43	73	0	0	0	NA	invasive ductal carcinomaa
6	66	0	0	0	NA	invasive carcinoma	44	56	0	0	0	3	invasive ductal carcinoma
7	41	0	0	0	3	invasive ductal carcinoma	45	42	0	0	NA	3	invasive ductal carcinoma
8	55	0	0	0	NA	invasive carcinoma	46	83	0	0	0	2	invasive ductal carcinoma
9	46	0	0	0	2	invasive ductal carcinomaa	47	34	0	0	0	3	invasive ductal carcinoma
10	49	0	0	0	NA	invasive ductal carcinomaa	48	37	0	0	0	3	invasive ductal carcinoma
11	43	0	0	0	3	invasive ductal carcinoma	49	40	0	0	0	3	metaplastic carcinoma
12	73	0	0	0	2	invasive ductal carcinoma	50	57	0	0	0	3	medullary carcinoma
13	56	0	0	0	3	malignant neoplasm	51	42	0	0	0	NA	invasive ductal carcinomaa
14	53	0	0	0	3	NA	52	72	0	0	0	3	invasive ductal carcinoma
15	37	0	0	0	3	invasive ductal carcinoma	53	28	0	0	0	3	NA
16	32	0	0	0	3	metaplastic carcinoma	54	32	0	0	0	3	invasive ductal carcinoma
17	34	0	0	0	NA	invasive ductal carcinomaa	55	33	0	0	0	NA	metastatic carcinoma
18	53	0	0	0	2	mixed invasive carcinoma	56	56	0	0	0	3	ductal carcinoma in situ
19	46	0	0	0	3	medullary invasive ductal carcinoma	57	54	0	0	0	3	invasive ductal carcinoma
20	33	0	0	0	3	invasive ductal carcinoma	58	50	0	0	0	3	invasive ductal carcinoma
21	44	0	0	0	3	invasive ductal carcinoma	59	60	0	0	0	3	invasive ductal carcinoma
22	52	0	0	0	2	ductal carcinoma in situ	60	68	0	0	0	2	invasive ductal carcinoma
23	68	0	0	0	3	invasive ductal carcinoma	61	74	0	0	0	3	invasive ductal carcinoma
24	38	0	0	0	NA	invasive ductal carcinomaa	62	40	0	0	0	3	invasive ductal carcinoma
25	40	0	0	0	3	invasive ductal carcinoma	63	54	0	0	0	3	invasive ductal carcinomaa
26	70	0	0	0	NA	invasive ductal carcinomaa	64	51	0	0	0	NA	metastatic
27	52	0	0	0	NA	metaplastic carcinoma	65	55	0	0	0	3	invasive ductal carcinoma
28	55	0	0	0	3	metastatic carcinoma	66	66	0	0	0	NA	NA
29	51	0	0	0	NA	invasive ductal carcinomaa	67	62	0	0	0	3	invasive ductal carcinoma
30	58	0	0	0	3	invasive carcinoma	68	42	0	0	0	2	NA
31	66	0	0	0	NA	invasive ductal carcinomaa	69	45	0	0	0	3	NA
32	46	0	0	0	3	invasive ductal carcinoma	70	60	0	0	0	3	invasive ductal carcinoma
33	50	0	0	0	3	invasive ductal carcinoma	71	41	0	0	0	3	invasive ductal carcinoma
34	53	0	0	0	3	metaplastic carcinoma	72	62	0	0	0	NA	invasive ductal carcinomaa
35	61	0	0	0	NA	invasive carcinoma	73	82	0	0	0	2	invasive ductal carcinoma
36	26	0	0	0	NA	pleomorphic carcinoma	74	45	0	0	0	3	invasive ductal carcinoma
37	56	0	0	0	2	invasive ductal carcinoma	75	56	0	0	0	3	NA
38	37	0	0	0	3	NA	76	42	0	0	0	NA	invasive ductal carcinomaa
							77	47	0	0	0	3	invasive ductal carcinoma

Supplementary Table 4. Sequences of primers used for 3'-UTR cloning and in qRT-PCR analysis, and list of antibodies used in Western blot or immunohistochemistry or immunofluorescence.

S4A. Sequences of forward and reverse primers used in qRT-PCR analysis.

Gene Symbol	Gene ID	Forward Primer (5' → 3')	Reverse Primer (5' → 3')
ACTB	60	CCAACCGCGAGAAGATGA	CCAGAGGCGTACAGGGATAG
GAPDH	2597	GCCCAATACGACCAAATCC	AGCCACATCGCTCAGACAC
HIF1A	3091	CCACAGGACAGTACAGGATG	TCAAGTCGTGCTGAATAATACC
LOX	4015	GGATACGGCACTGGCTACTT	GACGCCTGGATGTAGTAGGG
ITGA5	3678	GTCGGGGGCTTCAACTTAGAC	CCTGGCTGGCTGGTATTAGC
ITGA10	8515	GTGTGGATGCTTCATTCCAG	GCCATCCAAGACAATGACAA
ITGB5	3693	GGGAGTTTGCAAAGTTTCAGAG	TGTGCGTGGAGATAGGCTTT
FN1	2335	CTGGCCGAAAATACATTGTAAA	CCACAGTCGGGTCAGGAG

S4B. List of antibodies used in Western blot (WB), immunofluorescence (IF) and immunohistochemistry (IHC) analysis.

Antibody	Provider	Catalog number	WB dilution	IF dilution	IHC dilution
Alexa Fluor® 488 anti-mouse	Life Technologies	A-11001	-	1:200	-
Alexa Fluor® 488 anti-rabbit	Life Technologies	A-11034	-	1:200	-
Alexa Fluor® 647 anti-mouse	Life Technologies	A-31571	-	1:200	-
Alexa Fluor® 647 anti-rabbit	Life Technologies	A-31573	-	1:200	-
Alexa Fluor™ 488 Phalloidin	Life Technologies	A12379	-	1:400	-
Beta-actin	MP Biomedicals	691001	1:10000	-	-
CA9	Novus Biologicals	NB100-417	-	-	1:100
Cleaved Caspase -3	Cell Signaling Technology	9664	1:1000	1:200	1:400
Cleaved PARP	Cell Signaling Technology	5625	1:1000	-	-
Collagen I	Abcam	ab34710	-	1:200	-
FAK	Cell Signaling Technology	3285	1:1000	-	-
Flag	Sigma	F1804	1:1000	-	-
FN1	Santa Cruz	sc-81767	1:1000	1:200	-
GAPDH	Santa Cruz	sc-47724	1:10000	-	-
HIF1A	Abcam	51608	1:1000	-	1:300
HRP-coupled anti-mouse IgG	Cell Signaling Technology	7076S	1:10000	-	-
HRP-coupled anti-rabbit IgG	Cell Signaling Technology	7074S	1:10000	-	-
ITGA5	Sigma	HPA002642	1:1000	-	1:1000
Ki-67	ThermoFisher Scientific	MA5-14520	-	-	1:100
LOX	Abcam	174316	1:2000	-	1:400
LOX	Novus	NB100-2530	1:2000	-	1:400
LOXL2	Novus	NBP1-32954	-	-	1:200
phospho-FAK (Y397)	Abcam	39967	1:1000	-	-
phospho-Src (Y416)	Cell Signaling Technology	2101	1:1000	-	1:300
Src	Cell Signaling Technology	2108	1:1000	-	-

S4C. Sequences of forward and reverse primers used for 3'-UTR cloning.

Gene Symbol	Gene ID	Forward Primer (5' → 3')	Reverse Primer (5' → 3')
HIF1A	3091	CCGCTCGAGCATGTAGACTGCTGGGGC	ATTTGCGGCCGCCCACAGAAGATGTTTATTTGATGTAAC

LOX	4015	CCGCTCGAGTTCAATCCCTGAAATGTCTGC	ATTTGCGGCCGCCATAAAGCCAATGTCTGAGCA
ITGA5	3678	CCGCTCGAGCCCAATTTCAGACTCCCATTCCTG	ATTTGCGGCCGCGTTCTGGTCAGTGGGGGCAC

Supplementary Methods

Cell culture and reagents

Human TNBC cell lines, MDA-MB-231, MDA-MB-157, MDA-MB-436, HCC38, BT20 and HCC1143; ER+ cell lines, MCF-7, T47D and ZR-75-1; and HER2+ cell lines, SK-BR-3, JIMT-1, HCC1954; the human embryonic kidney cells, HEK293FT; mouse mammary cancer cell line, 4T1; and mouse embryonic fibroblast, NIH-3T3 were obtained from ATCC (Manassas, VA, USA). MDA-MB-231 cell line expressing luciferase and GFP (MDA-MB-231.Luc2GFP) that was used in the *in vivo* shLOX experiment was a kind gift from Dr. Dihua Yu (MD Anderson Cancer Center). Human foreskin fibroblast (HFF) cell line was a kind gift from Dr. Mythreye Karthikeyan (University of South Carolina). The original source of these modified cells is ATCC. All the cells were cultured in Dulbecco Modified Eagle Medium (Lonza, NJ, USA) supplemented with 50 U/ml penicillin/streptomycin, 1% non-essential amino acids and 10% fetal bovine serum (Lonza, NJ, USA). The media for ER+ cell lines were further supplemented with insulin (0.1 μg/ml). The cell lines were authenticated and tested for mycoplasma contamination regularly using MycoAlert mycoplasma detection kit (Lonza, NJ, USA). The cumulative culture length of cells between thawing and use in this study was less than 20 passages.

Transient transfection with miRNA mimics, siRNAs and reporters

Transfections were carried out by seeding cells in media containing no antibiotics, and changing media with growth factor reduced Optimem (Gibco, MA, USA) prior to transfection with Lipofectamine 2000 (Invitrogen, CA, USA) in 6-well or 96-well format[1,2]. miR-142-3p mimic and siLOX transfections were done at 40 nM concentrations, and RNA or protein was isolated after 48 hours of transfection. Reporter constructs carrying the 3'-UTRs of human *HIF1A* (NM_001530), *LOX* (NM_002317) and *ITGA5* (NM_002205) were used at a dose of 50 ng (for 96-well experiments) per well for the luciferase reporter assay experiments.

Cell viability assays

To assess the effects of LOX inhibition on doxorubicin response, cells were embedded in 0.5 mg/ml type I collagen in 96 well plates and were treated with BAPN (SantaCruz, TX, USA) or FAK inhibitor, PF-562271 (Selleckchem, TX, USA) or Src inhibitor, Saracatinib (Selleckchem, TX, USA) alone or in combination with doxorubicin for 36 hours. Cell viability was measured with 3D Cell Titer Glo Luminescent Cell Viability (Promega, WI, USA) assay according to the manufacturer's protocol using SoftMax Pro Software (Molecular Devices, CA, USA). To compare doxorubicin response between cells grown in the absence or presence of type I collagen, doxorubicin treatment was done for 72 hours. For the assessment of cell viability upon combination of doxorubicin with miR-142-3p overexpression, cells were transfected with 40 nM miR-142-3p mimic for 36 hours, and then embedded in 0.5 mg/ml type I collagen and treated with 500 nM doxorubicin for 48 hours.

Quantitative RT-PCR analysis

Total RNA was extracted from cultured cells and mice xenograft tumors using TRIsure (Bioline, Luckenwalde, Germany). For the latter, tumors were sliced into small pieces, a part of which was homogenized for complete lysis. Then, cDNA synthesis was performed using RevertAid RT Reverse Transcription Kit (Life Technologies, MA, USA) following manufacturer's protocol. Quantitative real-time PCR assay was carried out using Light Cycler 480 SYBR Green I Master kit in triplicates and data were collected with CFX Manager Software (Biorad, CA, USA). *GAPDH* and *HPRT* were used as housekeeping genes. The sequences of the qRT-PCR primers are provided in Supplementary Table 4a. For miRNA qRT-PCR experiments, *RNU44* and *RNU48* were used as housekeeping genes, and qRT-PCR was performed by TaqMan Real-Time PCR Assays (Thermo Fisher Scientific, MA, USA). For data analysis, $\Delta\Delta C_T$ method was utilized using Excel (Microsoft, WA, USA).

Western blotting

Protein isolation and Western blotting were done as previously described[1,2]. Briefly, proteins were extracted using RIPA lysis buffer with the addition of protease and phosphatase inhibitor cocktails, and protein concentrations were measured using the BCA Protein Assay Reagent Kit (Thermo Fisher Scientific, MA, USA). For extracting proteins from collagen-embedded cells, cells were treated with 1.5 mg/ml of collagenase solution containing phosphatase inhibitor cocktail for 10 minutes at 37 °C. Equal amounts of protein were separated using 8-10% SDS-PAGE gel. Separated proteins were transferred to PVDF membranes (Bio-Rad, CA, USA) using a Trans-Blot turbo transfer system (Bio-Rad, CA, USA) and incubated with primary antibodies (Supplementary Table 4b). Horseradish peroxidase-conjugated anti-mouse or anti-rabbit antibodies (Cell signaling Technology, MA, USA) were used as secondary antibodies, and signals were detected by enhanced chemiluminescence (Thermo Fisher Scientific, MA, USA). Images were acquired using Image Lab Software (Biorad, CA, USA).

Cell embedding in type I collagen matrix

Collagen solution was prepared at a concentration of 0.5-1 mg/ml from the rat tail collagen I (Corning, USA) with a neutralization step including the addition of 1N NaOH solution. Cell pellets were resuspended in neutralized collagen mix in PBS. While $8x10^3$ cells in 60 µl or in 30 µl were seeded into 96-well or µ-Slide 8-well glass bottom chambers, respectively; $3x10^5$ cells in 1 ml were seeded into 6 well plates. After 1 hour of incubation at room temperature, media was added on top of the solidified collagen, and the cells embedded in collagen were incubated at 37 °C for the desired amount of time. Drug treatments were done 12 hours after cell seeding.

Fibroblast-derived ECM

Generation of human foreskin fibroblasts (HFFs) derived ECM was performed by seeding HFFs onto coverslips with a cell density of $2x10^5$ cells per well and incubating at 37 °C for 6 days. Fibroblast cells were then removed using an extraction buffer containing 25 mM ammonium hydroxide and 0.5% Triton X-100. 20 ug/ml *DNAse I* treatment was done for 30 min to get rid of the remaining cell debris[3]. $2x10^5$

MDA-MB-231 cells treated with 10 mM BAPN or transfected with 40 nM miR-142-3p were seeded onto the extracted ECM and incubated for 72 hours at 37 °C followed by immunofluorescence staining of type I collagen and fibronectin.

Analysis of fibronectin fibril assembly by DOC lysis

DOC lysis assay was done by seeding MDA-MB-231 cells pretreated with vehicle or BAPN onto ECM, derived from HFF or NIH3T3 cells. After an additional 2 days of BAPN treatment, the matrix was solubilized by scraping in DOC lysis buffer that contains 2% Sodium Deoxycholate, 0.02 M Tris-HCl pH 8.8, 2 mM PMSF, 2 mM EDTA, 2 mM Iodoacetic Acid and 2 mM N-ethylmalemide. The solution was passed through 23G needle for 5 times. After shaking on ice for 30 min, samples were centrifuged at 21,130g for 30 min at +4. The supernatant was collected as the soluble fraction, mixed with 4X loading dye and boiled at 95 ºC for 5 min. The insoluble pellet was resuspended in 20 uL SDS lysis buffer, and then mixed with 4X loading dye followed by boiling at 95 ºC for 5 min. Samples were analyzed by Western blotting[4].

Immunofluorescence staining

Immunofluorescence staining of cells or type I collagen and/or fibronectin was done in μ-Slide 8-well glass bottom chambers (Ibidi, Germany). Cells were fixed with 4% paraformaldehyde for 20 min, permeabilized with 0.5% Triton X-100 for 10 min and blocked in 5% BSA-PBS. For staining of type I collagen and fibronectin, no permeabilization was done. Samples were incubated with primary and secondary Alexa Fluor 647 or 488-labeled antibodies for 1.5 hours at room temperature. Cells were also counterstained with DAPI for 5 min. Images were acquired using ZEN 2012 SP5 (Black) LSM 700 (Carl Zeiss, DE). Images were analyzed using the ImageJ software[5]. To quantify the cleaved Caspase-3 positive cells, the intensity from the red channel (the color for cleaved caspase 3) was adjusted to almost zero and the same intensity was applied to all other images (Supplementary Fig. 6d). Then, red signals that overlap with DAPI were quantified. A minimum of 200 cells were counted. As doxorubicin has intrinsic fluorescence with a

maximum excitation and emission wavelengths of 495 and 595 nM[6], respectively, we also acquired images from unstained, doxorubicin-treated cells, but detected no background fluorescence in green or red channels at the laser power and gain used for F-actin (green) or cleaved Caspase-3 visualizations.

For the analysis of ECM structures, images from 5-9 different areas in each biological replicate were acquired. For 3D collagen embedded samples, total collagen intensity of collagen was calculated as remaining "total green area" after the subtraction of the green intensity derived from intracellular collagen. Since fibronectin did not give any cell-derived signal in our system, fibronectin intensity was calculated as "total red area" of each sample. For co-localization quantification, a color threshold was applied to measure the spatial overlap between channels in identical pixel positions. To analyze the minimal distances of each nuclei (DAPI) to fibronectin, a 3D distance map was created. Calculated distances were clustered into 6 groups where "0" value represents the percentage of cells in contact with fibronectin.

Annexin V/DAPI staining

Cells were embedded in collagen I and treated with different drug combinations for 72 hours. After 72 hours, cells were washed once with PBS and the collagen matrix was degraded by applying 1.5 mg/ml collagenase for 5 minutes at 37 ºC. Cells were recovered from collagen matrix by pipetting several times, and the collagenase activity was inhibited by adding equal amount of media with 10% FBS. Cells were counted and 100,000 cells per group were collected, centrifuged and washed once with PBS. Cell pellet was dissolved in 100 µL Annexin V binding buffer and 1.5 µL of FITC-conjugated Annexin V was added. Cells were incubated at room temperature for 15 min in the dark followed by incubation with DAPI for an additional 5 minutes. Finally, cell solution was diluted in Annexin V binding buffer followed by data collection with BD FACSDiva software (BD, NJ, USA) and analysis with De Novo FCS Express software.

Hypoxia assay

Cells were seeded into 6-well plates and incubated overnight at 37 ºC in normoxic conditions. 24 hours later, plates were placed in Hypoxia Incubator Chamber (StemCell, MA, USA) in 1% Oxygen at 37 °C for

0-24 hours. After each time point, cells were collected by trypsinization and preserved for RNA and protein isolations.

Cloning the 3'-UTRs of the target genes

The construction of plasmids carrying the 3′-UTR sequences of human *HIF1A*, *LOX* and *ITGA5* genes was done by amplifying the 3′-UTR regions of *HIF1A*, *LOX* and *ITGA5* by PCR using genomic DNA of the MDA-MB-231 cell line and cloning downstream of the *Renilla* luciferase open reading frame in the psiCHECK-2 vector (Promega, WI, USA) using *XhoI* and *NotI* restriction sites[7]. The 3′-UTRs of *HIF1A*, *LOX* and *ITGA5* containing binding sites for miR-142-3p were amplified using primers listed in Supplementary Table 4c. These amplicons were ligated separately into psiCHECK-2 vector backbone.

Dual luciferase reporter assay

Dual-luciferase reporter assay was performed by seeding cells into 96-well plates and co-transfecting with miR-142-3p mimic and the 3'UTRs cloned into psiCHECK-2 vector. 24 hours after transfection, cells were lyzed, transferred to a white plate and the Dual Luciferase Reporter Assay (Promega, WI, USA) was performed according to manufacturer's instructions[7]. Luciferase activity was measured in Synergy HT microplate reader (BioTek, Vermont, USA), and the values were normalized to *firefly* luciferase activity.

LOX activity assay

LOX activity within cells or within tumors was measured using fluorometric LOX Activity Assay Kit (Abcam, MA, USA) according to manufacturer's instructions. Briefly, serially diluted replicates of tumor lysates or cell supernatants were incubated with reagent mix, and subsequently measured in fluorescence microplate reader at Ex/Em = 540/590 nm wavelength. Tumor lysates were prepared by sonicating tissue samples in extraction buffer (6 M urea, 10 mM Tris pH 7.4 and protease inhibitors) followed by centrifugation.

Collagen Assay

Collagen Assay from Abcam (ab222942) was performed following manufacturer's instructions. Briefly, fibroblast-derived ECM that was pre-incubated with BAPN or vehicle-treated cancer cells was solubilized in acid/pepsin mixture (0.5 M acetic acid and 0.1 mg/ml of pepsin) overnight at +4 ºC. The mixture is neutralized with 10 N NaOH and vortexed. Cell debris was removed by brief centrifugation, and the supernatant was collected as the soluble fraction. For HFF-derived ECM experiments, cell culture media was also collected as the soluble fraction. Then, the insoluble pellet was dissolved in 10 N NaOH at 120 ºC for 1 hour, followed by neutralization with 10 N HCl. For tumors samples a minimum of 10 mg tumor pieces were sonicated and hydrolyzed in 10 N NaOH for 1 hour at 120 ºC.

Lentiviral vector constructs and infections

TRIPZ inducible lentiviral non-silencing shRNA Control (RHS4743) and LOX shRNA vectors were obtained from Dharmacon. To produce viral particles, 6 µg of each of these vectors and 4.3 µl of trans-lentiviral packaging mix (Dharmacon, CO, USA) were used to co-transfect HEK293FT cells in 6-wells plate with $CaCl_2$ reagent (Dharmacon, CO, USA). 48 hours post-transfection, viral particles were harvested, and used to transduce MDA-MB-231 cells. 96 hours post-transduction, stably transfected cells were selected with 1 µg/ml of puromycin for 3-4 days.

Immunohistochemistry

Immunohistochemistry was performed on 3 µm cut sections using automated bond-max system (Leica microsystems GmbH, Wetzlar, Germany). Briefly, paraffin-embedded specimens were deparaffinized in Dewax solution and subjected to heat-mediated antigen-retrieval for 30 minutes at 90°C using Epitope Retrieval Solution 2 (EDTA-buffer pH 8.8) followed by peroxidase blocking for 5 minutes. Tissues were then incubated with the primary antibody (Supplementary Table 4b) for 15 minutes and with Post Primary Reagent for 8 minutes followed by washing with Bond Wash solution for another 6 minutes. Subsequently, tissues were incubated with Bond Polymer for 8 minutes and developed with DAB-Chromogen for 8

minutes followed by hematoxylin/eosin (H&E) counterstaining. Images were acquired using Olympus BX50 microscope. Immunoreactive scores were calculated as described previously[8].

For staining patient tumor samples, TMAs were constructed from formalin- fixed paraffin-embedded (FFPE) tissues of TNBC patients by the Advanced Tissue Arrayer (ATA100; Chemicon, Temecula, CA, USA) with the approval from the Non- Interventional Clinical Research Ethics Committee of Hacettepe University (approval no: 2020/02-40). Four- micrometer- thick sections were stained with H&E for histologic assessment of the samples in the TMAs. For histological analysis of each protein, the unstained slides were de-paraffinized and rehydrated gradually in decreasing concentrations of ethanol. Endogenous peroxidase activity was blocked with 3% H_2O_2 in methanol. Antigen retrieval was done by microwaving for 10 minutes in citrate buffer (pH 6.0) solution or 30 minutes in 10 mM EDTA buffer (pH 8.0). Slides were incubated with the primary antibodies for 1 hour at room temperature. The UltraVision Polyvalent (Rabbit-Mouse) HRP Kit (TP-125-HL, Thermo Scientific/LabVision), and the avidin-biotin peroxidase method was then used. The signals were developed using the chromogen, 3,3′-diaminobenzidine (DAB, TA-125-HD, Thermo Scientific/LabVision). Finally, samples were briefly counterstained with hematoxylin. The slides were dehydrated and prepared for microscopic examination.

Picrosirius Red staining

Tumor tissues obtained from mice were cut and fixed in a solution of 10% neutral buffered formalin and then embedded in paraffin. Slides were first deparaffinized by incubation at 60 °C for 10 min followed by two washes with xylene for 5 min each. Samples were hydrated by decreasing concentrations of ethyl alcohol (100%, 95%, 80% and 70%, respectively) and rinsed in distilled water. Hydrated sections were stained with Picrosirius Red Stain Kit (Polysciences, Inc.) according to manufacturer protocol and then, dehydrated with 100% ethanol. Sections were mounted after they were cleared in xylene for 5 minutes. Samples were analyzed using a bright field/polarized light microscope (Axiovert 200, Carl Zeiss, DE) with the Axiovision software (Carl Zeiss, DE). Images were converted to RGB, and the intensity of the red channel, representing collagen type I[9-11] was quantified using ImageJ software.

Measuring doxorubicin penetration *in vitro* and *in vivo*

As doxorubicin has intrinsic fluorescence with a maximum excitation and emission wavelengths of 495 and 595 nM, respectively[6], we quantified relative doxorubicin levels in cells embedded in collagen I *in vitro* by measuring the fluorescence intensity coming from doxorubicin or doxorubicin+BAPN treated cells at an excitation and emission wavelengths of 495 and 595 nM, respectively using a microplate reader. For measuring intratumoral doxorubicin levels, we embedded freshly excised tumors in optimum cutting temperature (OCT) compound, froze on dry ice, and stored at –80 °C. Cryostat sections were cut from each tumor, mounted on glass slides, and allowed to air-dry. Doxorubicin autofluorescence was detected using Zeiss LSM700 Confocal Microscopy with the 488 laser.

***In vivo* experiments**

In order to recapitulate *in vivo* resistant model for testing doxorubicin+BAPN combination once tumors develop doxorubicin resistance, MDA-MB-231 xenografts were developed and treated with doxorubicin (2.5 mg/kg) until tumors exhibit accelerated growth despite given therapy as described above. When the tumor volume reached 500 mm^3, they were randomly separated into two groups. One group continued to receive doxorubicin, while the other was treated with doxorubicin and BAPN (100 mg/kg, every other day, i.p.) combination. Survival was calculated using a predefined tumor volume cut-off of 1500 mm^3 or extensive toxicity as determined by a more than 20% decrease in body weight.

To test the role of miR-142-3p on hypoxia/LOX/integrin axis *in vivo*, inducible miR-142-3p overexpressing MDA-MB-231-Luc2-GFP cells were injected into nude mice. Doxycycline was given to induce miR-142-3p expression at 1 mg/ml in drinking water when the tumors reach 70-80 mm^3 for 6 days. Then, mice were sacrificed, and tumors were collected to examine protein expression *via* Western Blotting and miR-142-3q levels *via* qRT-PCR.

Patient data and pathway analyses

Patient data were retrieved from the NCBI GEO database (GSE16446[12], GSE19783[13], GSE21653[14], GSE22219[15], GSE22226[16], GSE25066[17], GSE58644[18], GSE31519 and GSE58812[19]) and from online survival analysis tool, KM-plotter[20], the Molecular Taxonomy of Breast Cancer International Consortium[21] project data from EMBL European Genome–Phenome Archive (http://www.ebi.ac.uk/ega/) with an accession number EGAS00000000122 and The Cancer Genome Atlas (TCGA) data from Chipbase V2 (rna.sysu.edu.cn/chipbase/). For pathway enrichment, the core analysis was performed at IPA platform using top differentially expressed (up- or down-regulated) mRNAs with an adjusted p-value cut-off of 0.05, from doxorubicin sensitive and resistant xenografts. GSEA was performed using gene sets related to hypoxia and integrin signaling downloaded from the GSEA website: http://software.broadinstitute.org/gsea/index.jsp.

Supplementary References

1. Saatci, O., *et al.* Targeting PLK1 overcomes T-DM1 resistance via CDK1-dependent phosphorylation and inactivation of Bcl-2/xL in HER2-positive breast cancer. *Oncogene* **37**, 2251-2269 (2018).
2. Mishra, R.R., *et al.* Reactivation of cAMP Pathway by PDE4D Inhibition Represents a Novel Druggable Axis for Overcoming Tamoxifen Resistance in ER-positive Breast Cancer. *Clinical cancer research : an official journal of the American Association for Cancer Research* **24**, 1987-2001 (2018).
3. Kaukonen, R., Jacquemet, G., Hamidi, H. & Ivaska, J. Cell-derived matrices for studying cell proliferation and directional migration in a complex 3D microenvironment. *Nature protocols* **12**, 2376-2390 (2017).
4. Varadaraj, A., Magdaleno, C. & Mythreye, K. Deoxycholate Fractionation of Fibronectin (FN) and Biotinylation Assay to Measure Recycled FN Fibrils in Epithelial Cells. *Bio Protoc* **8**(2018).
5. Schindelin, J., *et al.* Fiji: an open-source platform for biological-image analysis. *Nat Methods* **9**, 676-682 (2012).
6. Liang, J.Y., *et al.* Simple and rapid monitoring of doxorubicin using streptavidin-modified microparticle-based time-resolved fluorescence immunoassay. *Rsc Adv* **8**, 15621-15631 (2018).
7. Raza, U., *et al.* The miR-644a/CTBP1/p53 axis suppresses drug resistance by simultaneous inhibition of cell survival and epithelial-mesenchymal transition in breast cancer. *Oncotarget* **7**, 49859-49877 (2016).
8. Fedchenko, N. & Reifenrath, J. Different approaches for interpretation and reporting of immunohistochemistry analysis results in the bone tissue - a review. *Diagnostic pathology* **9**, 221 (2014).
9. Cox, T.R., *et al.* LOX-mediated collagen crosslinking is responsible for fibrosis-enhanced metastasis. *Cancer research* **73**, 1721-1732 (2013).
10. Junqueira, L.C., Cossermelli, W. & Brentani, R. Differential staining of collagens type I, II and III by Sirius Red and polarization microscopy. *Arch Histol Jpn* **41**, 267-274 (1978).
11. Raj, Y., *et al.* Evaluation of the Nature of Collagen Fibers in KCOT, Dentigerous Cyst and Ameloblastoma using Picrosirius Red Stain - A Comparative Study. *J Clin Diagn Res* **9**, ZC01-04 (2015).
12. Desmedt, C., *et al.* Multifactorial approach to predicting resistance to anthracyclines. *Journal of clinical oncology : official journal of the American Society of Clinical Oncology* **29**, 1578-1586 (2011).
13. Enerly, E., *et al.* miRNA-mRNA integrated analysis reveals roles for miRNAs in primary breast tumors. *PloS one* **6**, e16915 (2011).
14. Sabatier, R., *et al.* A gene expression signature identifies two prognostic subgroups of basal breast cancer. *Breast cancer research and treatment* **126**, 407-420 (2011).
15. Buffa, F.M., *et al.* microRNA-associated progression pathways and potential therapeutic targets identified by integrated mRNA and microRNA expression profiling in breast cancer. *Cancer research* **71**, 5635-5645 (2011).
16. Esserman, L.J., *et al.* Chemotherapy response and recurrence-free survival in neoadjuvant breast cancer depends on biomarker profiles: results from the I-SPY 1 TRIAL (CALGB 150007/150012; ACRIN 6657). *Breast cancer research and treatment* **132**, 1049-1062 (2012).
17. Hatzis, C., *et al.* A genomic predictor of response and survival following taxane-anthracycline chemotherapy for invasive breast cancer. *Jama* **305**, 1873-1881 (2011).
18. Tofigh, A., *et al.* The prognostic ease and difficulty of invasive breast carcinoma. *Cell reports* **9**, 129-142 (2014).
19. Jezequel, P., *et al.* Gene-expression molecular subtyping of triple-negative breast cancer tumours: importance of immune response. *Breast cancer research : BCR* **17**, 43 (2015).

20. Gyorffy, B., *et al.* An online survival analysis tool to rapidly assess the effect of 22,277 genes on breast cancer prognosis using microarray data of 1,809 patients. *Breast cancer research and treatment* **123**, 725-731 (2010).
21. Curtis, C., *et al.* The genomic and transcriptomic architecture of 2,000 breast tumours reveals novel subgroups. *Nature* **486**, 346-352 (2012).
