# Supplementary material for: Targeting lysyl oxidase (LOX) overcomes chemotherapy resistance in triple negative breast cancer
Source: Nat Commun. 2020 May 15;11:2416. doi: 10.1038/s41467-020-16199-4 (PMC7229173; doi:10.1038/s41467-020-16199-4)
Supplement: Supplementary file 3 — Description of Additional Supplementary Files [file 41467_2020_16199_MOESM3_ESM.pdf]

Description of Additional Supplementary Files

File Name: Supplementary Data 1
Description: List of genes in doxorubicin resistance gene signature. Top deregulated genes between doxorubicin sensitive vs. resistant xenografts developed *in vivo* (n = 441)
