# Supplementary material for: Targeting lysyl oxidase (LOX) overcomes chemotherapy resistance in triple negative breast cancer
Source: Nat Commun. 2020 May 15;11:2416. doi: 10.1038/s41467-020-16199-4 (PMC7229173; doi:10.1038/s41467-020-16199-4)
Supplement: Supplementary file 5 — Reporting Summary [file 41467_2020_16199_MOESM5_ESM.pdf]

nature research

Corresponding author(s): Ozgur Sahin

Last updated by author(s): Apr 9, 2020

Reporting Summary

Nature Research wishes to improve the reproducibility of the work that we publish. This form provides structure for consistency and transparency in reporting. For further information on Nature Research policies, see Authors & Referees and the Editorial Policy Checklist.

Statistics

For all statistical analyses, confirm that the following items are present in the figure legend, table legend, main text, or Methods section.

n/a	Confirmed	
☐	☒	The exact sample size (*n*) for each experimental group/condition, given as a discrete number and unit of measurement
☐	☒	A statement on whether measurements were taken from distinct samples or whether the same sample was measured repeatedly
☐	☒	The statistical test(s) used AND whether they are one- or two-sided<br>*Only common tests should be described solely by name; describe more complex techniques in the Methods section.*
☒	☐	A description of all covariates tested
☐	☒	A description of any assumptions or corrections, such as tests of normality and adjustment for multiple comparisons
☐	☒	A full description of the statistical parameters including central tendency (e.g. means) or other basic estimates (e.g. regression coefficient) AND variation (e.g. standard deviation) or associated estimates of uncertainty (e.g. confidence intervals)
☐	☒	For null hypothesis testing, the test statistic (e.g. *F*, *t*, *r*) with confidence intervals, effect sizes, degrees of freedom and *P* value noted<br>*Give P values as exact values whenever suitable.*
☒	☐	For Bayesian analysis, information on the choice of priors and Markov chain Monte Carlo settings
☒	☐	For hierarchical and complex designs, identification of the appropriate level for tests and full reporting of outcomes
☐	☒	Estimates of effect sizes (e.g. Cohen's *d*, Pearson's *r*), indicating how they were calculated

Our web collection on statistics for biologists contains articles on many of the points above.

Software and code

Policy information about availability of computer code

Data collection	CFX Manager Software version 3.0 (Biorad), SoftMax Pro Software version 7.0.2 (Molecular Devices), Image Lab Software version 6.0.0 (Biorad), ZEN 2012 SP5 (Black) LSM 700 14.0.0.0 Software (Carl Zeiss), Axiovision version 4.0 (Carl Zeiss), Lumina III In Vivo Imaging System (Perkin Elmer), LSR II (BD), BD FACSDiva version 8.0, GSEA version 2.2.3.
Data analysis	Excel 2015 (Microsoft), GraphPad Prism software version 8.0, ImageJ version 1.52p (FIJI), SPSS Software version 22 (IBM), Living Image software version 4.5.4, De Novo Software: FCS Express version 6, TopHat version 2.1.0, HTSeq framework 0.11.2, Bioconductor edgeR package version 3.14.0, IPA Spring Release (2016)

For manuscripts utilizing custom algorithms or software that are central to the research but not yet described in published literature, software must be made available to editors/reviewers. We strongly encourage code deposition in a community repository (e.g. GitHub). See the Nature Research guidelines for submitting code & software for further information.

Data

Policy information about availability of data

All manuscripts must include a data availability statement. This statement should provide the following information, where applicable:

- Accession codes, unique identifiers, or web links for publicly available datasets
- A list of figures that have associated raw data
- A description of any restrictions on data availability

The RNA-Seq data has been uploaded to the Sequence Read Archive (SRA) of NCBI with the submission ID: SUB6918779 (https://www.ncbi.nlm.nih.gov/bioproject/PRJNA607780). Data presented on Figures 1d, f, 2b and Supplementary Figure 2b, 2c, 4a, 4b were generated by analyzing the data available under the accession number GSE58812 from GEO depository (https://www.ncbi.nlm.nih.gov/geo/). Data presented on Figures 1h, 1i, 1k, 1l, 2o and Supplementary Figure 1d, 1e, 2a, 2d were generated by analyzing the KM Plotter database (http://kmplot.com/analysis/). Data presented on Figures 7c, 7d and Supplementary Figure 1a, 12d were generated by analyzing the METABRIC data from EMBL European Genome–Phenome Archive (http://www.ebi.ac.uk/ega/) with an accession number EGAS00000000122. GSEA gene sets were downloaded from the GSEA MSigDB Collections website: https://www.gsea-msigdb.org/gsea/msigdb/collections.jsp. Data presented on Figures 7b and Supplementary Figure 12a, b, c were generated by analyzing the data available under the accession number GSE19783 from NCBI. Data presented on Supplementary Figure 1b, 5a were generated by analyzing the data available under the accession number GSE31519 from NCBI. Data presented on Supplementary Figure 5b, 5c, 5d, 5e were generated by analyzing the data available under the accession number GSE25066 from NCBI. Data presented on Figure 2h was generated by analyzing the data available under the accession numbers GSE16446, GSE19783, GSE21653, GSE22219, GSE22226, GSE25066, GSE58644 from NCBI and under METABRIC datasets and The Cancer Genome Atlas (TCGA) data. The source data underlying Figs. 1a-h, 1j-k; 2a-e, 2g-o, 2q; 3a-g, 3i, 3k-q; 4a-e, 4g-j, 4l, 4n-p, 4r; 5a, 5c-e, 5g, 5i, 5k-l; 6a, 6c, 6e-f, 6h-i, 6k, 6m, 6o, 6q, 6s; 7b-c, 7e-j, 7l-m, 7p and Supplementary Figs. 1a-c; 2a-c; 3a-b; 4c-d; 5c-e; 6b, 6f; 7a-d; 8a, 8c; 9a, 9c; 10a-c, 10e-f; 11b-f; 12e are provided as a Source Data file. All the other data supporting the findings of this study are available within the article and its supplementary information files and from the corresponding author upon reasonable request. A reporting summary for this article is available as a Supplementary Information file.

Field-specific reporting

Please select the one below that is the best fit for your research. If you are not sure, read the appropriate sections before making your selection.

☒ Life sciences ☐ Behavioural & social sciences ☐ Ecological, evolutionary & environmental sciences

For a reference copy of the document with all sections, see nature.com/documents/nr-reporting-summary-flat.pdf

Life sciences study design

All studies must disclose on these points even when the disclosure is negative.

Sample size	No sample size calculations was performed. For in vitro experiments, sample sizes were chosen based on experience from previous experiments to reach statistically relevant results. Sample sizes for in vivo experiments were determined based on previous studies (ref. 29, 30).
Data exclusions	Technical replicates identified as outliers during the in vitro studies (identified by Graphpad) and poor quality paraffin-embedded blocks of patient tissue samples that didn't allow getting sections for further staining were excluded.No samples were excluded from RNA-seq analysis.
Replication	Experiments were repeated two to three times and reproducible results were obtained.
Randomization	In vitro samples were randomly allocated into different treatment groups. For in vivo experiments, mice were randomly allocated into different treatment groups.
Blinding	Investigators were not blinded while allocating mice into groups, during treatment, data collection or analysis because the sample names contained treatment information.

Reporting for specific materials, systems and methods

We require information from authors about some types of materials, experimental systems and methods used in many studies. Here, indicate whether each material, system or method listed is relevant to your study. If you are not sure if a list item applies to your research, read the appropriate section before selecting a response.

Materials & experimental systems

n/a	Involved in the study	
☐	☒	Antibodies
☐	☒	Eukaryotic cell lines
☒	☐	Palaeontology
☐	☒	Animals and other organisms
☐	☒	Human research participants
☒	☐	Clinical data

Methods

n/a	Involved in the study	
☒	☐	ChIP-seq
☐	☒	Flow cytometry
☒	☐	MRI-based neuroimaging

Antibodies

Antibodies used

All antibodies were purchased as follows with their vendors and catalogue numbers:
Beta-actin, MP Biomedicals, 691001, Lot#=127M4866V
HIF1A, Abcam, 51608, Lot#=GR244245-30
LOX, Abcam, 174316, Lot#=GR211601-18
LOX, Novus, NB100-2530, Lot#=D2
ITGA5, Sigma, HPA002642, Lot#=B74062
phospho-FAK (Y397), Abcam, 39967, Lot#= GR240190-4
phospho-Src (Y416), Cell Signaling Technology, 2101, Lot#=20
Src, Cell Signaling Technology, 2108, Lot#=9
FAK, Cell Signaling Technology, 3285, Lot#=9
Ki-67, ThermoFisher Scientific, MA5-14520
Cleaved Caspase-3, Cell Signaling Technology, 9664, Lot#=21
Cleaved PARP, Cell Signaling Technology, 5625, Lot#=13
CA9, Novus Biologicals, NB100-417, Lot#=AH-1
FN1, Santa Cruz , sc-81767, Lot#=F1913
Collagen I, Abcam, ab34710, Lot#=GR3297275-1
GAPDH, Santa Cruz, sc-47724, Lot#=C3117
Flag, Sigma F1804, Lot#=SLBX2256
LOXL2, Novus,NBP1-32954, Lot#=43236
HRP-conjugated anti-rabbit and anti-mouse secondary antibodies (Cell Signaling Technology, 7074S and 7076S). Alexa Fluor-labelled secondary antibodies are all from Life Technologies: Alexa Fluor® 488 anti-mouse, A11001; Alexa Fluor® 488 anti-rabbit, A11034; Alexa Fluor® 647 anti-mouse, A31571; Alexa Fluor® 647 anti-rabbit, A31573; Alexa Fluor 488 Phalloidin, A12379

Validation

All antibodies are commercially available and validated in the literature as cited on the manifacturer's websites, as well as by the datasheet they provide. Below are the validation data and application notes from suppliers' websites:

Beta-actin, MP Biomedicals, 691001: Immunoblots demonstrate specificity directed towards all six known vertebrate isoactins. HIF1A, Abcam, 51608: Tested in human samples for WB, IF, IHC and IP. LOX, Abcam, 174316: Tested in human and mouse samples for WB, IF, IHC and IP. LOX, Novus, NB100-2530: Tested in human and mouse samples for WB, IF and IHC. ITGA5, Sigma, HPA002642: Tested in human and mouse samples for WB and IHC. phospho-FAK (Y397), Abcam, 39967: Tested in human, mouse and rat brain tissue lysates for WB and in SK-N-SH cell line for IF. phospho-Src (Y416), Cell Signaling Technology, 2101: Tested in human and mouse samples for WB. Src, Cell Signaling Technology, 2108: Tested in human and mouse samples for WB, IP, IF and flow cytometry. FAK, Cell Signaling Technology, 3285: Tested in human and mouse samples for WB, IP and IHC. Cleaved Caspase-3, Cell Signaling Technology, 9664: Tested in human and mouse samples for WB, IHC, IP, IF and flow cytometry. Cleaved PARP, Cell Signaling Technology, 5625: Tested in human and mouse samples for WB, IHC, IP, IF and flow cytometry. CA9, Novus Biologicals, NB100-417: Tested in several human cancer tissue for IHC. FN1, Santa Cruz , sc-81767: Tested in human samples for WB and immunoperoxidase staining. Collagen I, Abcam, ab34710: Validated in Horse bronchial fibroblast cells for IF. Also validated for IHC and WB with human and mouse samples. GAPDH, Santa Cruz, sc-47724: Tested in humand and mouse samples for WB. Flag, Sigma F1804: . Optimized for single banded detection of FLAG fusion proteins in mammalian, plant, and bacterial expression systems. LOXL2, Novus,NBP1-32954: Knockout validated with WB using LOXL2 konckout Hela cell extracts. Also validated for IHC using mouse kidney and human carcinoma tissues.

Eukaryotic cell lines

Policy information about cell lines

Cell line source(s)	Human TNBC cell lines, MDA-MB-231, MDA-MB-157, MDA-MB-436, HCC38, BT20 and HCC1143. ER+ cell lines, MCF-7, T47D and ZR-75-1; and HER2+ cell lines, SK-BR-3, JIMT-1, HCC1954; humen embryonic kidney cell line, HEK293FT, mouse mammary cancer cell line, 4T1 and mouse embryonic fibroblast, NIH-3T3 were obtained from ATCC (Manassas, VA, USA). MDA-MB-231 cell line expressing luciferase and GFP (MDA-MB-231.Luc2GFP) that was used in the in vivo shLOX experiment was a kind gift from Dr. Dihua Yu (MD Anderson Cancer Center). Human foreskin fibroblast (HFF) cell line was a kind gift from Dr. Mythreye Karthikeyan (University of South Carolina). The commercial source of the original MDA-MB-231 and HFF cells is ATCC.
Authentication	The cell lines were authenticated using STR Genotyping.
Mycoplasma contamination	All cell lines were tested as negative for mycoplasma contamination using MycoAlert mycoplasma detection kit (Lonza, NJ, USA).
Commonly misidentified lines (See ICLAC register)	There are no misidentified cell lines in this study.

Animals and other organisms

Policy information about studies involving animals; ARRIVE guidelines recommended for reporting animal research

Laboratory animals	Mice used in the study were all female. The strains used were athymic nu/nu, Balb/c and NSG mice. All mice were 6-8 weeks old. All mice were maintained under a temperature-controlled environment with a 12-hour light/dark cycle and received a standard diet and water ad libitum.
Wild animals	There are no wild animals used in this study.
Field-collected samples	There are no field-collected samples used in this study.
Ethics oversight	All animal experiments were approved by the Animal Ethics Committee of Bilkent University or the Institutional Animal Care and Use Committee of University of South Carolina.

Note that full information on the approval of the study protocol must also be provided in the manuscript.

Human research participants

Policy information about studies involving human research participants

Population characteristics	All patient characteristics are summarized in Supplementary Table 3. All patients were white females. Furthermore, the IHC staining scores from these patient samples are provided in the Source Data. The TNBC organoid, F149T was generated from breast cancer tissue of a black female patient.
Recruitment	The primary tumor samples from 77 TNBC patients who were diagnosed with breast cancer between 2006 to 2015 at Hacettepe University School of Medicine, Ankara, Turkey and treated with chemotherapy (35% adjuvant anthracycline-based therapy, 43% anthracyclines in combination with taxanes and 22% other chemotherapy agents) were used for histological analyses of LOX protein. To generate TNBC patient organoids, breast cancer surgical patients were consented under an IRB approved protocol for the USC-Palmetto Health Biorepository. The organoid (F149T) was selected based on growth characteristics in order to be able to conduct experiments in a reasonable time frame.
Ethics oversight	The study was approved by the Non-Interventional Clinical Research Ethics Committee of Hacettepe University (approval no: 2020/02-40). For the generation of patient organoids, breast cancer surgical patients were consented under an IRB approved protocol for the USC-Palmetto Health Biorepository.

Note that full information on the approval of the study protocol must also be provided in the manuscript.

Flow Cytometry

Plots

Confirm that:

- ☒ The axis labels state the marker and fluorochrome used (e.g. CD4-FITC).
- ☒ The axis scales are clearly visible. Include numbers along axes only for bottom left plot of group (a 'group' is an analysis of identical markers).
- ☒ All plots are contour plots with outliers or pseudocolor plots.
- ☒ A numerical value for number of cells or percentage (with statistics) is provided.

Methodology

Sample preparation	Cells were embedded in collagen I and treated with different drug combinations for 72 hours. After 72 hours, cells were washed once with PBS and the collagen matrix was degraded by applying 1.5 mg/ml collagenase for 5 minutes at 37 ºC. Cells were recovered from collagen matrix by pipetting several times, and the collagenase activity was inhibited by adding equal amount of media with 10% FBS. Cells were counted and 100,000 cells per group were collected, centrifuged and washed once with PBS. Cell pellet was dissolved in 100 uL Annexin V binding buffer and 1.5 uL of FITC-conjugated Annexin V was added. Cells were

	incubated at room temperature for 15 min in the dark followed by incubation with DAPI for an additional 5 min. Finally, cell solution was diluted in Annexin V binding buffer followed by analysis with flow cytometry.
Instrument	The instrument used for data collection was the LSR II Flow cytometry (BD).
Software	Data collection was performed with the BD FACSDiva software and analysis was done using the De Novo Flow cytometry Software
Cell population abundance	The percentage viability was around 85%. Loss of cell viability was due to the collaganase treatment that was applied to get rid of the collagen fibers. However, a very low dose was applied with a very short incubation time to minimize cell death.
Gating strategy	Gating parameters were set using different samples including untreated and only doxorubicin-treated cells, and cells incubated only with Annexin V or DAPI antibodies.

☒ Tick this box to confirm that a figure exemplifying the gating strategy is provided in the Supplementary Information.
